# Variant-Specific Kinetics of SARS-CoV-2 Anti-Nucleocapsid Antibodies and Household Transmission in Families During Anchestral, Alpha, Delta and Omicron Periods

**DOI:** 10.3390/life16030470

**Published:** 2026-03-13

**Authors:** Filippos Filippatos, Elizabeth-Barbara Tatsi, Vassiliki Syriopoulou, Athanasios Michos

**Affiliations:** Infectious Diseases and Chemotherapy Research Laboratory, First Department of Pediatrics, Medical School, National and Kapodistrian University of Athens, “Aghia Sophia” Children’s Hospital, 11527 Athens, Greece; etatsi@med.uoa.gr (E.-B.T.); vsyriop@med.uoa.gr (V.S.); amichos@med.uoa.gr (A.M.)

**Keywords:** SARS-CoV-2, nucleocapsid antibody, pediatric immunity, household transmission, Delta, Omicron

## Abstract

To investigate SARS-CoV-2 antibody kinetics and household transmission, infected children along with their families were tested for anti-nucleocapsid antibodies at 1, 3, 6, 9 and 12 months post-SARS-CoV-2 infection during the Ancestral, Alpha, Delta, and Omicron waves. We prospectively included SARS-CoV-2 acute infected children (n = 189). After household recruitment (n = 76 households), the total study population was 228 children and 105 adults. The median age (IQR) of children and adults was 96 (115) months and 504 (96) months, respectively. Anti-nucleocapsid (anti-N) COI (cut-off index) titers peaked at three months post-infection and declined thereafter (*p*-value < 0.001), and 89.2% remained seropositive at 12 months. Children displayed significantly higher anti-N COI titers than adults during the Delta (*p*-value: 0.018) and Omicron (*p*-value: 0.047) periods. Household contact anti-N positivity (evidence of infection) was associated with pediatric index cases (aOR: 1.61, 95% CI: 1.11–2.35; *p*-value: 0.013) and elevated early anti-N COI titers (aOR: 1.24 per log_10_ unit, 95% CI: 1.05–1.48; *p*-value: 0.011). Higher secondary attacks were detected in Delta (aOR: 2.12, 95% CI: 1.19–3.77; *p*-value: 0.011) and Omicron (aOR: 2.75, 95% CI: 1.44–5.25; *p*-value: 0.002) compared to Ancestral. Waning of SARS-CoV-2 anti-N titers was faster in secondary cases (aHR: 1.62, 95% CI: 1.01–2.59; *p*-value: 0.047, Cox model) and during Omicron infection (aHR: 1.74 vs. Ancestral, 95% CI: 1.08–2.79; *p*-value: 0.023). In contrast, waning was slower in SARS-CoV-2 cases with higher baseline anti-N COI titers (aHR: 0.77, 95% CI: 0.64–0.93; *p*-value: 0.011). These findings demonstrate variant-specific, age-dependent antibody kinetics, emphasizing that pediatric index cases were associated with higher odds of household infection.

## 1. Introduction

Since the beginning of the COVID-19 pandemic, it has been known that children represent an important reservoir of SARS-CoV-2 infection in many regions [1,2]. Over 750 million confirmed infections worldwide have generated complex immune landscapes shaped by age, variants, vaccination, and repeated exposures [3]. Early studies suggested that SARS-CoV-2 anti-nucleocapsid (anti-N) antibodies wane with a half-life of 100–150 days but persist in ~80% of adults at one year [4,5]. However, these studies were not performed in the pediatric population and five years after the pandemic onset, data on pediatric SARS-CoV-2 antibody kinetics after natural infection remain limited [6].

Since antibodies against SARS-CoV-2 spike protein (anti-S) are produced after either natural infection or immunization, anti-N antibodies remain the best serological marker of prior infection [6,7,8]. Pediatric immune responses are distinct, featuring more robust germinal center activity and broader B-cell repertoires [9]. With low early vaccination and changing exposure patterns due to shifting public health measures [10], children’s role in household transmission increased during later waves. Household cohorts offer powerful settings for longitudinal analysis [11,12], yet few studies cover the Ancestral-to-Omicron timeline with child-validated assays. Greece’s four variant waves [13] enable a unique comparison of anti-N kinetics across ages and variants.

This study aims to characterize 12-month anti-N antibody kinetics across four SARS-CoV-2 variant waves (Ancestral, Alpha, Delta, Omicron), compare titer magnitude and waning between children and adults and assess household transmission.

## 2. Materials and Methods

### 2.1. Study Design and Participants

This is an observational, population-based prospective cohort study performed at “Aghia Sophia” Children’s Hospital, which is the largest (750-bed) pediatric hospital in Greece and was also a pediatric COVID-19 reference center for the Athens metropolitan area.

The research time frame was divided into four different periods, each corresponding to the predominant circulation of a major SARS-CoV-2 variant of concern in Greece between 2020 and 2022: Ancestral (1 May–31 December 2020), Alpha (1 January–30 July 2021), Delta (1 August–31 December 2021) and Omicron (1 January–31 December 2022).

Study participants included children aged 0–16 years who had confirmed SARS-CoV-2 infection (rapid antigen test or RT-PCR), as well as their household members (both children and adults), to assess intra-household transmission. Transmission was defined as serologically confirmed SARS-CoV-2 infection in household contacts, as indicated by anti-N antibody positivity. Predominance periods were defined using Greek National Public Health Organization (NPHO) National Genomic Surveillance Network reports and ECDC weekly country-level variant distribution data [14,15]. To assess possible misclassification during periods of variant replacement, SARS-CoV-2 infections occurring within transition windows around each period boundary were prospectively excluded from the study. For the purposes of the current study, the term “children” is used throughout the manuscript to refer to participants aged <18 years and the term “pediatric” is used only when referring to institutional or clinical contexts.

Household contacts were classified as infected if they had (i) a documented positive RT-PCR/rapid antigen test and/or (ii) anti-N seropositivity (cut-off index—COI ≥ 1) at follow-up. Where available, dates of RT-PCR/rapid antigen positivity were used to anchor infections temporally relative to the household episode. We defined household contact anti-N positivity as evidence of SARS-CoV-2 infection. Seroconversion was defined as the transition from anti-N seropositive (COI ≥ 1.0) to seronegative (COI < 1.0) at follow-up after a prior documented seropositive measurement. An index case was defined as the first household member with documented SARS-CoV-2 infection, based on the earliest available positive RT-PCR or rapid antigen test date. When diagnostic test dates were unavailable, the symptom onset date was used as the temporal anchor. A secondary case was defined as any other household member with evidence of SARS-CoV-2 infection (positive RT-PCR/rapid antigen test and/or anti-N seropositivity) occurring after the index case or within a temporally compatible window for household exposure. In households where multiple members tested positive on the same date or where temporal sequencing could not be reliably determined, cases were classified conservatively based on the earliest documented symptom onset.

The exclusion criteria were the following: 1. Children and adults with confirmed SARS-CoV-2 prior infection, verified by SARS-CoV-2 antigen or molecular diagnostic testing, 2. Children with underlying diseases or immunocompromised conditions or prior blood transfusion or intravenous IVIG administration.

A detailed form including demographic (age and sex) and COVID-19 history data was completed for all study participants. Children were categorized based on their age as neonates and infants (0–≤1 year old), toddlers (>1 years), preschool-age children (>4 years), school-age children (>6 years), and adolescents (>12).

The investigation was conducted for both children and adult participants at five different time points—one month (1 m), three months (3 m), six months (6 m), nine months (9 m), and 12 months (12 m)—following confirmed SARS-CoV-2 infection and across all SARS-CoV-2 variant predominance periods. SARS-CoV-2 vaccination was recorded as receipt of BNT162b2 (Pfizer-BioNTech, New York, NY, USA) with a maximum of two doses (no booster doses).

### 2.2. SARS-CoV-2 Antibody Detection

Serological measurements were performed using the Elecsys^®^ Anti-SARS-CoV-2 assay (Roche Diagnostics, Basel, Switzerland) on a cobas e 411 analyzer. The assay provided a qualitative interpretation (reactive/non-reactive) and a numeric cut-off (COI) and signal (sample)/signal (cut-off); samples with a COI ≥ 1.0 were considered reactive [16]. Although the assay is intended for qualitative detection and COI is not an absolute antibody concentration, COI represents a continuous immunoreactivity signal and was used as a semi-quantitative anti-N COI titer for within-assay comparisons.

### 2.3. Statistical Analysis

Statistical significance was set at a *p*-value ≤ 0.05 level. All statistical analyses were conducted using R version 4.3.2 (R Foundation for Statistical Computing, Vienna, Austria), with additional descriptive summaries performed in IBM SPSS Statistics version 25.0 (IBM Corp., Armonk, NY, USA). Figures were generated using GraphPad Prism 10 (GraphPad Software, San Diego, CA, USA). Descriptive summaries are reported as median [IQR] with 95% confidence intervals for the median. CIs for medians were obtained using a nonparametric bootstrap (10,000 resamples), applying a cluster bootstrap at the household level (households resampled with replacement; all eligible individuals within selected households included) to account for within-household correlation. Between-group differences are reported as effect sizes with 95% CIs (e.g., difference in medians for nonparametric comparisons, or model-based contrasts for mixed models), with CIs derived from the same cluster bootstrap procedure (percentile method, 2.5–97.5th percentiles).

Kurtosis and skewness were used for the assumption of normality assessment, and the Shapiro–Wilk and Kolmogorov–Smirnov tests were used for its confirmation. Following the assumption of normality assessment, median and interquartile range (IQR) were applied to quantitative variables. The qualitative variables were described using absolute and relative frequencies (%). Differences between qualitative variables were assessed using the Chi-square test. Differences between qualitative and quantitative variables involved the application of two nonparametric tests: the Mann–Whitney test (qualitative with 2 categories) and the Kruskal–Wallis test (qualitative with >2 categories). The Spearman r correlation coefficient was used to evaluate correlations between quantitative variables. For longitudinal kinetics and regression models, we treated COI as an assay-specific semi-quantitative antibody signal and analyzed log10(COI) to reduce skewness.

Multiple linear regression models were used to estimate SARS-CoV-2 anti-nucleocapsid antibody titers following natural infection in children and adults, treating antibody titer as a continuous dependent variable and study period (variant wave), age, and sex as independent variables. Adjusted odds ratios (aORs) from multilevel logistic models describe the relative odds of a binary event (household transmission), whereas adjusted hazard ratios (aHRs) from Cox models represent time-to-event effects (seroreversion). Both are presented with 95% confidence intervals (CIs), and two-sided *p*-values ≤ 0.05 are considered statistically significant. Kaplan–Meier curves compared time-to-loss of seropositivity. Anti-Ν COI titers were also analyzed longitudinally at the individual measurement level (each participant’s measurement at 1, 3, 6, 9, and 12 months post-infection). To account for the non-independence of repeated measurements and household clustering, we used linear mixed-effects models with log10(COI) as the dependent variable. Models included fixed effects for follow-up time (categorical), variant predominance period, age group (child vs. adult), and sex, with prespecified interaction terms (time × variant and age group × variant). Since vaccination dates were not available, the time since the last dose at infection could not be calculated, and dose count was coded as 0 vs. 1–2 doses. We included random intercepts for the participants and households (participants nested within households). Models were fitted using restricted maximum likelihood and incorporated all available measurements under a missing-at-random assumption.

Confounders were selected a priori based on biological plausibility and published evidence regarding determinants of antibody response and infection dynamics. The primary adjustment set included age group, sex, variant period, vaccination history (number of doses), and baseline antibody level (for seroconversion analyses). Household size was additionally included in household infection models. Covariates were retained irrespective of statistical significance to reduce residual confounding, and multicollinearity was assessed using variance inflation factors. No clinically relevant multicollinearity (VIF > 5) was observed.

### 2.4. Ethical Approval

The study protocol was conducted in accordance with the 1964 Declaration of Helsinki and was approved by the Scientific and Bioethics Committee of “Aghia Sophia” Children’s Hospital (No. 25609, 25 November 2020). Written informed consent was obtained from all adults and children for participation in this study.

## 3. Results

### 3.1. Study Population

A total of 189 pediatric index cases with confirmed SARS-CoV-2 acute infection were enrolled. Among these, 2.6% (5/189) of children were seronegative for SARS-CoV-2 anti-N and were excluded from the kinetics study.

**Figure 1:**
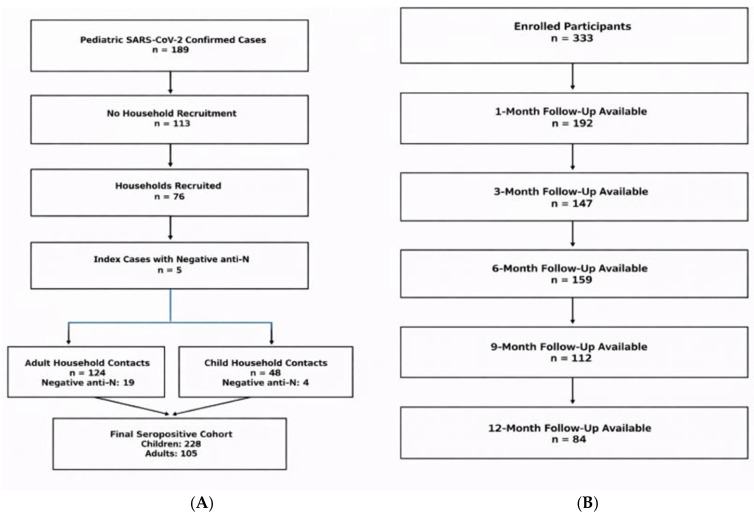
(**A**) A flowchart of pediatric SARS-CoV-2-confirmed index cases and household recruitment. (**B**) A CONSORT-style flow diagram showing follow-up completion at 1, 3, 6, 9, and 12 months after infection. Numbers represent participants with available anti-N measurements at each time point.

Household participation was feasible in 76/189 children, and an additional 124 adults and 44 children family members were included. Serological testing for SARS-CoV-2 Anti-N was negative for 15.3% (19/124) of adult members and 8.3% (4/48) of child members. Consequently, the number of household members with detectable SARS-CoV-2 Anti-N was 105 adults and 44 children. When combined with the pediatric index cases, this yielded a total of 333 participants—228 children and 105 adults with evidence of prior SARS-CoV-2 infection (Figure 1A). Anti-N measurements were available for 192 participants at 1 month, 147 at 3 months, 159 at 6 months, 112 at 9 months, and 84 at 12 months post-infection (Figure 1B).

A total of 333 participants were included in the study—228/333 (68.5%) children and 105/333 (31.5%) adults. The median age (IQR) of children and adults was 96 (115) months and 504 (96) months, respectively. The age distribution for children was 43/228 (18.9%) aged 0–1 years, 34/228 (15.0%) aged >1 years, 14/228 (6.2%) aged >4 years, 83/228 (36.6%) aged >6 years and 53/228 (23.3%) aged >12 years.

Participants were recruited during periods of predominant circulation of different SARS-CoV-2 variants: 46/333 (13.8%) during the Ancestral strain period, 87/333 (26.1%) during the Alpha period, 135/333 (40.5%) during the Delta period, and 65/333 (19.5%) during the Omicron period. Vaccination status at the first follow-up (1-month visit) was documented for 63/105 adults and 180/228 children. Among those with recorded status, 46/63 adults (73.0%) and 16/180 children (8.9%) were vaccinated. Vaccination in children was higher in adolescents (10/27; 37.0% of adolescents with recorded status) compared with school-age children (6/83; 7.2%), while no vaccinated cases were recorded among infants/toddlers/preschool children at the 1-month visit. Vaccination prevalence increased across later variant periods, being highest during Omicron (children: 8/27; 29.6% with recorded status; adults: 9/11; 81.8%). Characteristics of the study population are presented in Table 1.

### 3.2. Anti-N Seropositivity and Persistence Through 12 Months

By 12 months post-infection, 297/333 participants (89.2%) remained seropositive (COI ≥ 1). At 12 months after SARS-CoV-2 infection, 77/84 participants (91.7%) remained anti-N seropositive. Seropositivity was 50/54 (92.6%) in children and 27/30 (90.0%) in adults (*p*-value > 0.05). Among children, seropositivity at 12 months was: infants (0–1 year): 6/6 (100%); toddlers (>1 years): 4/6 (66.7%); preschool children (>6 years): 6/6 (100%); school-age children (>6 years): 27/29 (93.1%); and adolescents (>12 years): 7/7 (100%) (*p*-value > 0.05).

The median anti-N COI titer at 12 months was 14.8 COI (IQR: 5.03–42.69; bootstrap 95% CI: 8.76–22.91) for all participants; 14.15 COI (IQR: 5.09–38.66; 95% CI: 8.87–24.46) for index cases; and 15.49 COI (IQR: 4.35–61.68; 95% CI: 7.79–42.65) for secondary cases (*p*-value: 0.048). The SARS-CoV-2 natural infection median anti-N COI (IQR) and bootstrap 95% CIs at 1 m, 3 m, 6 m, 9 m and 12 m in the total study population, children and adults are presented in Table 2.

### 3.3. Longitudinal Anti-N COI Titers Kinetics by Age Group and Variant Period

In the 228 children, the maximum and minimum SARS-CoV-2 median (ΙQR) anti-N COI titers were detected at 3 m and 12 m after natural infection, respectively (*p*-value < 0.001). During the whole study period, no significant differences were detected regarding SARS-CoV-2 median anti-N COI titers between males and females at 1 m (*p*-value: 0.321), 3 m (*p*-value: 0.911), 6 m (*p*-value: 0.644), 9 m (*p*-value: 0.497) and 12 m (*p*-value: 0.519) after SARS-CoV-2 natural infection. In females, SARS-CoV-2 median anti-N COI titers were significantly higher at 3 m compared to 1 m after SARS-CoV-2 natural infection (*p*-value: 0.043). In males, SARS-CoV-2 median anti-N COI titers were significantly higher at 3 m compared to 1 m after SARS-CoV-2 natural infection (*p*-value: 0.024).

No statistically significant differences were detected in median SARS-CoV-2 anti-N COI titers between the different age groups at all the time points. SARS-CoV-2 median anti-N COI titers per time point significantly differed during the Ancestral wave in both children (*p*-value: 0.004) and adults (*p*-value: 0.017) (Table 2). In children, no other significant differences were detected (*p*-value: 0.176 in Alpha, *p*-value: 0.457 in Delta and *p*-value: 0.263 in Omicron). In adults, no other significant differences were detected (*p*-value: 0.652 in Alpha, *p*-value: 0.425 in Delta and *p*-value: 0.871 in Omicron).

**Figure 2:**
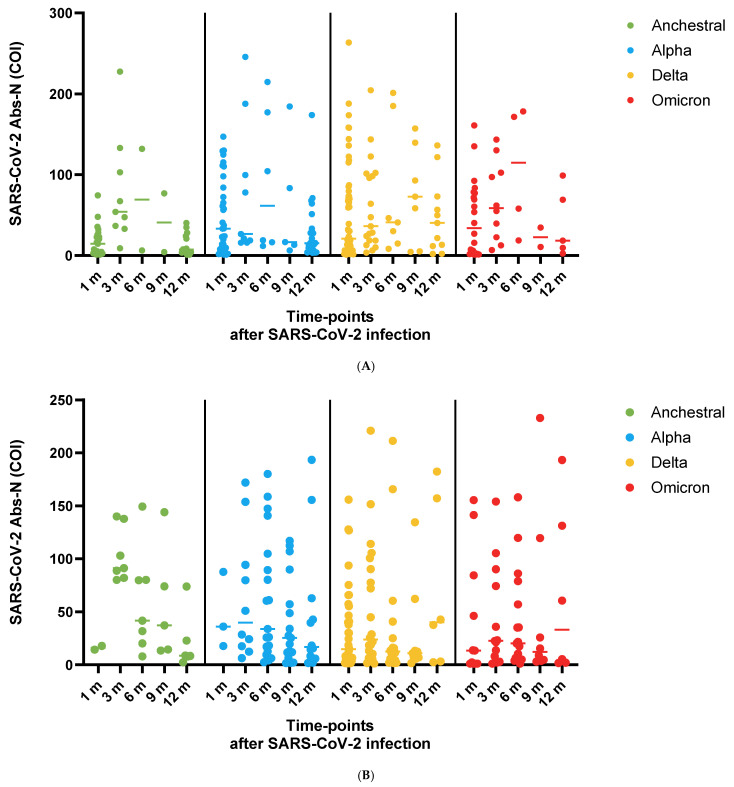
(**A**) Median anti-N cut-off index (COI) values in children at 1, 3, 6, 9, and 12 months following natural SARS-CoV-2 infection, stratified by variant predominance period (Ancestral, Alpha, Delta, Omicron). (**B**) Corresponding anti-N COI values in adults. Points represent median anti-N values; error bars represent interquartile ranges. Statistical comparisons between children and adults at each time point are indicated as follows: Anti-N seropositivity was defined as COI ≥ 1.0.

No significant differences were detected in SARS-CoV-2 median anti-N COI titers per variant in children (*p*-value: 0.082 in 1 m, *p*-value: 0.815 in 3 m, *p*-value: 0.806 in 6 m, *p*-value: 0.747 in 9 m and *p*-value: 0.115 in 12 m, respectively) (Figure 2A and Table 2). No significant differences were detected in SARS-CoV-2 median anti-N COI titers per variant in adults (*p*-value: 0.669 in 1 m, *p*-value: 0.127 in 3 m, *p*-value: 0.077 in 6 m, *p*-value: 0.37 in 9 m and *p*-value: 0.857 in 12 m, respectively) (Figure 2B and Table 2).

SARS-CoV-2 median anti-N COI titers significantly differed between children and adults in the Ancestral (*p*-value: 0.002) and Delta (*p*-value: 0.018) periods but not in Alpha (*p*-value: 0.588) and Omicron (*p*-value: 0.206). During Delta, SARS-CoV-2 median anti-N COI titers were higher in children than in adults at 6 m (*p*-value: 0.047). Children had significantly higher SARS-CoV-2 median anti-N COI titers than adults at 6 m (*p*-value: 0.024) and not at other time points. SARS-CoV-2 median (IQR) anti-N COI titers were significantly lower in adult males than in females during the Ancestral (*p*-value: 0.026) and Delta (*p*-value: 0.017) periods but lower during Alpha (*p*-value < 0.001). Among the 105 adults, no significant differences were detected regarding SARS-CoV-2 median anti-N COI titers per time point (*p*-value: 0.11). During the whole study period, no significant differences were detected regarding SARS-CoV-2 median Abs titers between males and females at any time point. In adults, a statistically significant negative correlation between SARS-CoV-2 median anti-N COI titers and age was detected only at 3 m (Spearman r_0_: −0.448, *p*-value: 0.022) and not at other time points.

A statistically significant model was revealed for children (*p*-value: 0.031, R^2^ = 0.018), with a significant predictor being the SARS-CoV-2 variant parameter (β = 8.124, *p*-value: 0.018). The results of the multiple linear regression analysis for children are presented in Supplementary Table S1. A statistically significant model was revealed for adults during the Ancestral period (*p*-value: 0.046, R^2^: 0.161), with a significant predictor being sex (β = 36.099, *p*-value: 0.05) (Supplementary Table S1), during the Alpha period (*p*-value: 0.026, R^2^ = 0.088), with a significant predictor being sex (β = −34.613, *p*-value: 0.011) (Supplementary Table S1) and during the Delta period (*p*-value: 0.027, R^2^: 0.058), with a significant predictor being sex (β = 28.103, *p*-value: 0.01) (Supplementary Table S1). No significant multiple linear regression model was found for the Omicron predominance period in adults (*p*-value: 0.694).

Using longitudinal mixed-effects models with random intercepts for participant and household, anti-N COI titers varied significantly over time (*p*-value < 0.001) with a peak at 3 months and a decline thereafter, and kinetics differed by variant (time × variant *p*-value < 0.001). Children exhibited higher anti-N COI titers than adults during the Delta (*p*-value: 0.024) and Omicron (*p*-value: 0.032) periods, whereas no significant child–adult differences were observed during Ancestral or Alpha.

### 3.4. Household Contact Anti-N Positivity as Evidence of Infection and Associated Factors

Among household contacts, anti-N positivity (evidence of infection) was observed in 44/48 (91.7%) child contacts and 105/124 (84.7%) adult contacts (*p*-value: 0.32). Household contact was associated with pediatric index cases (aOR 1.61, 95% CI 1.11–2.35; *p*-value: 0.013) and elevated early anti-N COI titers (aOR 1.24 per log_10_ unit, 95% CI 1.05–1.48; *p*-value: 0.011) but not with household size (aOR 1.13, 95% CI 0.98–1.3; *p*-value: 0.091). Compared with Ancestral, Delta (aOR 2.12, 95% CI 1.19–3.77; *p*-value: 0.011) and Omicron (aOR 2.75, 95% CI 1.44–5.25; *p*-value: 0.002) waves showed higher household contact infection odds (anti-N positivity). No significant differences were detected between Alpha and Ancestral (aOR 1.32, 95% CI 0.71–2.44; *p*-value: 0.382).

In the longitudinal mixed-effects model (log10 COI outcome) adjusted for follow-up month, variant period, age group, and sex, participants vaccinated at the 1-month visit had lower anti-N COI titers compared to unvaccinated participants (β = −0.420 log10 COI, 95% CI −0.648 to −0.193, *p*-value < 0.001), corresponding to an approximately 0.38-fold lower COI titers (95% CI 0.22–0.64).

### 3.5. Seroconversion over 12 Months

**Figure 3:**
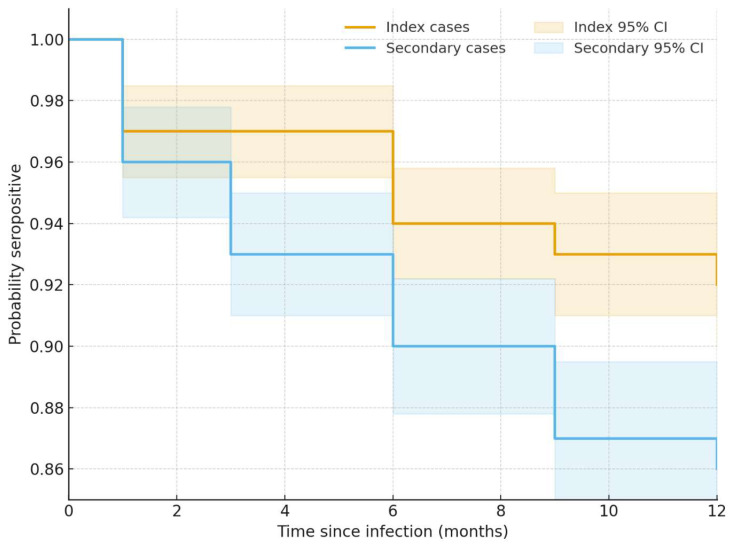
Kaplan–Meier curves illustrate the probability of maintaining anti-N seropositivity (COI ≥ 1.0) over 12 months among SARS-CoV-2–infected children, stratified by case status (index—orange line vs. secondary—blue line). Shaded areas represent 95% confidence intervals. Time zero corresponds to the date of documented infection. Participants were censored at their last-available serological measurement if they remained seropositive. Index cases retain antibodies longer; the log-rank difference is significant (*p*-value: 0.045).

Kaplan–Meier analysis among infected children showed faster loss of anti-N seropositivity in secondary cases compared with index cases (log-rank *p* = 0.045) (Figure 3). In Cox proportional hazards models, secondary case status was associated with faster seroconversion (aHR 1.62, 95% CI 1.01–2.62). Higher baseline anti-N COI titers were associated with slower seroconversion (aHR 0.77 per log_10_ unit, 95% CI 0.64–0.92). Compared with Ancestral, Omicron-period infection was associated with faster seroconversion (aHR 1.74, 95% CI 1.08–2.80), while Alpha and Delta did not significantly differ from Ancestral. Age group and sex were not associated with seroconversion (Table 3).

## 4. Discussion

In this 12-month prospective cohort study, we aimed to investigate SARS-CoV-2 anti-N antibody kinetics across four SARS-CoV-2 variant predominance periods. We found that anti-N remained detectable in most participants one year after SARS-CoV-2 infection. Titers peaked at 3 months and declined thereafter, and children showed higher titers than adults at specific time points. Nearly all participants developed detectable anti-N with durability varying by variant and age, and peak titers were highest in pediatric index cases, particularly during the Delta and Omicron waves.

By 12 months, approximately 90% of participants remained anti-N seropositive, with no significant seropositivity differences between children and adults. Méndez-Echevarría et al. showed 86% (48/56) pediatric IgG positivity at 6 months, while we extended follow-up to 12 months and showed sustained seropositivity in most children at one year [13]. Renk et al. showed seropositivity at 11–12 months of 96.2% in children and 82.9% in adults, respectively. These data agree regarding high durability but differ in the magnitude of the child–adult gap, potentially reflecting differences in variant eras, exposure patterns, and cohort composition [17]. Loesche et al. showed an 11.6% seronegativity by 12 months using Roche Elecsys anti-N [18]. Haynes et al. showed a post-peak half-life of approximately 120 days and projected that > 90% would remain detectable beyond 1 year and 50% beyond 2 years [19]. These results support high one-year detectability of anti-N across all age groups.

Household transmission was independently associated with pediatric index cases, higher early anti-N titers and infection during Delta/Omicron, while seroconversion occurred more rapidly in secondary cases and in Omicron-period infections. Madewell et al. showed pooled household secondary attack rates rising across variant eras (18.9% early/wild-type, 29.7% Delta, 42.7% Omicron), while we showed higher transmission odds in later waves (Delta aOR 2.12 vs. Ancestral; Omicron aOR 2.75 vs. Ancestral) [20]. These data are compatible with our findings and demonstrate increased household spread in Delta and Omicron compared with earlier periods. Lyngse et al. showed higher household secondary attack rates for Omicron than Delta in Denmark (29% vs. 21%), while we showed higher transmission odds in Omicron than Delta relative to Ancestral. These data support increased within-household spread during the Omicron era [21].

Our findings suggest that, within households, pediatric index cases were associated with higher odds of household infection. Paul et al. showed that 27.3% of 6280 households with pediatric index cases had secondary transmission and that younger pediatric index cases had higher odds of transmission (e.g., OR 1.43 for 0–3 vs. 14–17 years) [22]. In contrast, Viner et al. showed no significant difference in transmissibility from children/young people compared with adults (pooled relative transmissibility 0.92, 95% CI 0.68–1.26) [10]. These differences could possibly be attributed to different period recruitment (early and later variants) and that in the present study, serology-defined outcome captured mild or asymptomatic secondary infections.

In this cohort, anti-N titers peaked at 3 months post-infection for both children and adults. Haynes et al. showed a post-peak half-life of approximately 120 days [19]. Loesche et al. showed model-dependent longer-term seroconversion estimates approaching 50% by 24 months, while we showed no abrupt collapse in seropositivity within the first year [18]. Even though these are not directly comparable in time frame, they are suggestive that longer-term estimates are sensitive to model choice and assay thresholds. Seow et al. showed anti-N peaking around 60–90 days and declining by approximately 0.5 log10 by 300 days using the same Elecsys platform, while we showed a peak at 3 months with gradual waning and high 12-month detectability [23]. These data agree on peak timing and direction of change, and the slower anti-N decline through 9–12 months is compatible with the higher baseline titers and younger case mix in our cohort.

Van Elslande et al. showed earlier seronegativity using an IgG indirect anti-N assay (50% seronegative at 273–327 days), while we showed substantially higher 12-month seropositivity with a total antibody Elecsys format [24]. These data agree that anti-N wanes over time but underscore strong assay dependence of time to seronegativity. Ahmad et al. reported persistence of anti-N IgG in 89% of previously infected healthcare workers at 12 months (with 10% seronegative at month 12), and a peak with higher median anti-N IgG around 6 months before waning (median 32.9 COI at 6 months falling to 9.8 COI at 12 months), which is consistent with our observed post-3-month decline and 90% 12-month detectability in the adult population [25].

When comparing children and adults, we showed that children had significantly higher titers than adults at 6 months and that child–adult differences were variant-dependent, with significant differences detected in the Ancestral and Delta periods. Renk et al. showed higher antibody levels and lower seroconversion rates in children than adults through 11–12 months, while we showed a smaller but directionally compatible child advantage (higher titers in selected periods and slightly higher 12-month seropositivity in children). These data support that pediatric humoral responses can be robust, but our results indicate that the magnitude of this effect may vary by variant era and sampling time.

This study showed faster anti-N decline in secondary cases and during Omicron and that higher baseline titers were associated with higher seropositivity rates at 12 months. Loesche et al. showed 11% seronegativity by 12 months and emphasized baseline antibody titers as a key driver of subsequent threshold crossing [18], while we showed nearly identical one-year seronegativity rates and quantified baseline titers as protective in Cox models. Haynes et al. showed slow decay with an antibody half-life of 120 days and that at 12 months, seronegative participants tended to have low peak signals [19]. These data agree with our study in both magnitude and mechanism, suggesting that low baseline titers may predict faster waning.

Multiple studies indicate that anti-nucleocapsid (anti-N) assays can be less sensitive for identifying prior infection in vaccinated individuals. In the blinded phase of the randomized mRNA-1273 efficacy trial, Follmann et al. showed that anti-N seroconversion using the Roche Elecsys anti-N assay after PCR-confirmed COVID-19 occurred in 40% of vaccine breakthrough cases versus 93% of placebo recipients, highlighting a substantial decrease in detectable anti-N responses after infection in vaccinated persons [26]. Similarly, Allen et al. reported that among fully vaccinated healthcare workers with PCR-confirmed breakthrough infection, only 26% had detectable anti-N antibodies despite sampling around 52 days after PCR positivity, compared with 82% anti-N positivity among participants with prior PCR-confirmed infection overall [27]. Dhakal et al. found lower anti-N IgG responses in both plasma and oral fluid after infection in vaccinated mild COVID-19 patients compared with infections occurring before vaccination or in unvaccinated individuals, supporting the concern that anti-N–based surveillance may underdetermine infections in cohorts with vaccinated participants [28].

Since Elecsys anti-N reports an assay-specific COI and the magnitude above the cut-off is not a direct measure of total antibody amount, absolute COI values and waning rates are not directly comparable across platforms or manufacturers. The WHO National Institute of Biological Standards and Control (NIBSC) supports harmonization by defining BAU/mL but explicitly notes that this assists comparison only among assays detecting the same immunoglobulin class with the same specificity (e.g., anti-RBD IgG vs. anti-N IgM are not directly comparable) and that units must not be used interchangeably with other assays [29]. Longitudinal studies show that measured antibody waning depends on the test used, including the immunoglobulin class (e.g., IgG vs. total antibody) and antigen target (N vs. S/RBD). In a healthcare-worker follow-up, anti-N IgG declined markedly faster than anti-S IgG, underscoring why apparent waning rates can differ across studies that use different targets and assay formats [30].

Persistent anti-N seropositivity primarily serves as evidence of prior SARS-CoV-2 infection. However, anti-N COI values are assay-specific, and the Elecsys anti-N assay is intended for qualitative detection. It remains uncertain whether detectable antibodies confer protective immunity and protect against future infection or severe disease. Although recent cohort studies have reported that higher pre-reinfection anti-N levels are associated with lower reinfection risk, this likely reflects prior infection burden and hybrid immunity rather than a direct neutralizing activity, which is mainly linked to anti-spike responses [31,32,33]. Therefore, our findings are best interpreted as describing the durability of an infection-induced serological signal over time rather than demonstrating durable protective immunity.

Limitations of the present research include the exclusive assessment of SARS-CoV-2 anti-N without neutralizing data, a relatively modest sample size, incomplete documentation of socioeconomic and clinical parameters, and a follow-up restricted only to 12 months. Since vaccination dates were unavailable and booster doses were not administered, we could not model time since last dose or booster effects. Participants were recruited from a tertiary hospital setting, which may limit generalizability. As a result, antibody kinetics and household infection patterns observed here may not fully reflect those in the general population. The Roche Elecsys anti-SARS-CoV-2 assay reports a cut-off index (COI) and is intended for qualitative interpretation (reactive ≥ 1.0). We measured total antibodies (anti-N) using a commercial electrochemiluminescence assay, but no neutralization testing was performed. The study period spanned multiple variant waves and increasing vaccine coverage, which may have affected anti-N kinetics in study participants. Although COI was analyzed as a semi-quantitative within-assay signal, it does not represent an absolute antibody concentration and is not directly comparable across platforms. However, a strength of this study is the inclusion of child–parent household clusters through four different SARS-CoV-2 variant predominance periods (Ancestral, Alpha, Delta, Omicron) with standardized 12-month anti-N follow-up.

## 5. Conclusions

In this variant-specific antibody kinetics and transmission study of SARS-CoV-2 during four different variant periods, natural infection induced long-lasting anti-nucleocapsid immunity in most participants, yet magnitude and durability were strongly modulated by variant and transmission role. Children infected during Delta and Omicron waves mounted significantly higher and more persistent anti-N responses than adults, reversing the pattern seen in early waves. Pediatric index cases and elevated initial titers were each independently associated with household spread, underscoring the growing epidemiological significance of school-age infections. Omicron infection, however, was associated with both attenuated titers and accelerated seronegativity, signaling that future serosurveillance must account for variant-specific assay reactivity. These insights refine our understanding of post-infection SARS-CoV-2 immunity and support age- and variant-tailored public-health strategies.

## Figures and Tables

**Table 1:** Baseline characteristics of study participants.

Characteristic	Total (N = 333)	Children (n = 228)	Adults (n = 105)
Participants, n	333	228	105
Age, median (IQR), months	120 (132)	96 (115)	504 (96)
Male sex, n (%)	167 (50.2)	120 (52.6)	47 (44.8)
Variant period, n (%)			
Ancestral	46 (13.8)	37 (16.2)	9 (8.6)
Alpha	87 (26.1)	65 (28.5)	22 (21.0)
Delta	135 (40.5)	88 (38.6)	47 (44.8)
Omicron	65 (19.5)	38 (16.7)	27 (25.7)

**Table 2:** SARS-CoV-2 natural infection anti-N COI median (IQR) with bootstrap 95% confidence intervals for the median at 1, 3, 6, 9 and 12 months in the total study population, children and adults. Cut-off index (COI) values of ≥1 were positive.

		Ancestral n = 46	Alpha n = 87	Delta n = 135	Omicron n = 65	*p*-Value
Total study population (N = 333)	1 m (n = 192)	14.33 (19.16) [4.89–20.44]	33.09 (69.58) [14.01–57.56]	18.55 (61.47) [13.26–30.67]	15.53 (75.30) [2.72–60.51]	0.094
Total study population (N = 333)	3 m (n = 147)	95.48 (85.07) [53.86–116.40]	34.22 (123.80) [22.56–87.09]	47.42 (87.91) [23.99–77.56]	39.34 (74.82) [21.98–70.11]	0.164
Total study population (N = 333)	6 m (n = 159)	71.95 (81.12) [20.79–80.25]	28.46 (87.56) [18.60–61.11]	32.59 (71.17) [16.22–46.14]	33.33 (61.77) [19.90–51.86]	0.244
Total study population (N = 333)	9 m (n = 112)	19.23 (66.97) [6.44–72.03]	19.54 (46.09) [12.91–31.18]	58.53 (86.19) [13.23–72.59]	27.04 (54.83) [8.95–44.31]	0.805
Total study population (N = 333)	12 m (n = 84)	7.57 (17.52) [3.94–14.75]	15.39 (33.77) [9.14–27.63]	40.20 (61.48) [11.57–73.05]	39.37 (84.97) [5.45–100.14]	0.111
Total study population (N = 333)	*p*-value	<0.001	0.031	0.090	0.524	
		**Ancestral** **n = 37**	**Alpha** **n = 65**	**Delta** **n = 88**	**Omicron** **n = 38**	***p*-Value**
Children (N = 228)	1 m (n = 140)	14.29 (20.03) [4.37–22.65]	33.09 (69.58) [13.71–61.02]	20.66 (66.34) [13.68–49.15]	33.62 (73.61) [3.99–71.74]	0.082
Children (N = 228)	3 m (n = 95)	103.00 (85.70) [47.30–133.00]	38.87 (130.93) [21.20–139.60]	56.86 (79.99) [30.16–95.84]	53.04 (74.42) [22.56–96.98]	0.815
Children (N = 228)	6 m (n = 98)	73.53 (108.08) [13.45–127.40]	28.46 (84.47) [18.33–73.21]	44.10 (86.80) [30.02–79.28]	36.76 (65.24) [29.47–63.28]	0.806
Children (N = 228)	9 m (n = 73)	16.72 (67.73) [3.83–72.03]	17.97 (45.01) [12.61–41.65]	64.47 (85.82) [19.37–88.59]	38.90 (55.46) [17.32–184.20]	0.747
Children (N = 228)	12 m (n = 54)	6.02 (14.20) [3.33–20.75]	15.10 (23.52) [9.14–27.63]	40.20 (52.47) [11.57–73.05]	18.17 (59.67) [2.44–98.85]	0.115
Children (N = 228)	*p*-value	<0.004	0.176	0.457	0.263	
		**Ancestral** **n = 9**	**Alpha** **n = 23**	**Delta** **n = 47**	**Omicron** **n = 26**	***p*** **-Value**
Adults (N = 105)	1 m (n = 52)	16.06 (1.73) [14.33–17.79]	26.82 (35.48) [0.46–87.61]	13.59 (48.75) [4.95–40.08]	13.33 (64.80) [0.09–84.43]	0.669
Adults (N = 105)	3 m (n = 52)	88.84 (39.36) [35.40–103.00]	28.49 (72.15) [12.42–94.37]	18.74 (84.01) [9.24–77.56]	22.62 (66.19) [8.09–74.28]	0.127
Adults (N = 105)	6 m (n = 61)	41.61 (54.01) [20.21–80.13]	29.96 (81.41) [14.84–84.79]	11.24 (20.89) [4.21–25.10]	20.17 (51.17) [5.89–57.06]	0.077
Adults (N = 105)	9 m (n = 39)	25.81 (51.23) [6.72–109.02]	22.47 (47.40) [8.96–52.93]	10.86 (31.46) [5.65–62.20]	12.17 (44.75) [3.75–119.60]	0.37
Adults (N = 105)	12 m (n = 30)	8.53 (11.45) [5.04–48.41]	15.49 (36.79) [5.86–42.65]	40.20 (116.72) [2.88–169.75]	60.56 (125.85) [0.76–193.20]	0.857
Adults (N = 105)	*p*-value	0.017	0.652	0.425	0.871	

**Table 3:** Possibility of seroconversion for children and adults (N = 333) at 12 months from seropositive SARS-CoV-2 anti-N to seronegative.

Predictor (Reference)	aHR	95% CI	*p*-Value
Secondary case (index)	1.62	1.01–2.62	0.047
Log10 titre at 1 mo (per unit)	0.77	0.64–0.92	0.011
Child (adult)	0.91	0.64–1.29	0.580
Male (female)	1.05	0.75–1.47	0.790
Alpha wave (Ancestral)	0.93	0.49–1.78	0.830
Delta wave (Ancestral)	1.25	0.83–1.87	0.280
Omicron wave (Ancestral)	1.74	1.08–2.80	0.023

## Data Availability

The original contributions presented in this study are included in the article/Supplementary Materials. Further inquiries can be directed to the corresponding author.

## Supplementary Materials

The following supporting information can be downloaded at: https://www.mdpi.com/article/10.3390/life16030470/s1, Table S1: Multiple linear regression model analysis of 228 SARS-CoV-2 seropositive children and 105 SARS-CoV-2 seropositive adults for the whole study period (May–December 2022) involving SARS-CoV-2 natural infection antibody titers as dependent variable and months after SARS-CoV-2 infection, sex and SARS-CoV-2 variant period as independent variables
