# Supplementary material for: Variant-Specific Kinetics of SARS-CoV-2 Anti-Nucleocapsid Antibodies and Household Transmission in Families During Anchestral, Alpha, Delta and Omicron Periods
Source: Life (Basel). 2026 Mar 13;16(3):470. doi: 10.3390/life16030470 (PMC13027493; doi:10.3390/life16030470)
Supplement: Supplementary file 1 [file life-16-00470-s001.zip › life-4159013-supplementary.pdf]

Table S1. Multiple linear regression model analysis of 228 SARS-CoV-2 seropositive children and 105 SARS-CoV-2 seropositive adults for the whole study period (May 2020 – December 2022) involving SARS-CoV-2 natural infection antibody titers as dependent variable and months after SARS-CoV-2 infection, sex and SARS-CoV-2 variant period as independent variables. Statistically significant differences (*P*-value <0.05) are marked in bold.

		**Unstandardized Coefficients**		**Standardized Coefficients**	***P*-value**	**95% Confidence Interval**	
		**β**	**Std. Error**	**Beta**		**Lower Bound**	**Upper Bound**
**Children (n=228)**	**Constant**	16.889	14.608		0.249	-11.871	45.648
	**Month after SARS-CoV-2 infection**	-0.803	2.100	-0.023	0.703	-4.938	3.332
	**Sex**	9.317	6.639	0.084	0.162	-3.753	22.386
	**Variant period**	8.124	3.424	0.143	**0.018**	1.383	14.866
**Adults (n=105)**	**Constant**	23.698	40.381		0.583	-58.644	107.039
	**Month after SARS-CoV-2 infection**	-9.980	6.384	-0.282	0.131	-23.157	3.196
	**Sex**	36.099	18.313	0.356	0.06	-1.698	73.890
**Adults Alpha period (n=22)**	**Constant**	123.116	28.751		<0.001	65.546	180.687
	**Month after SARS-CoV-2 infection**	-5.223	5.870	-0.324	0.377	-16.973	6.527
	**Sex**	-34.613	13.185	-0.324	0.011	-61.005	-8.221
**Adults Delta period (n=47)**	**Constant**	-12.659	20.158		0.532	-52.718	27.401
	**Month after SARS-CoV-2 infection**	-0.803	2.100	0.112	0.277	-3.857	13.282
	**Sex**	28.103	10.715	0.27	0.01	6.81	49.397
